# Impact of Bioactive Ingredients on the Fecal Excretion of Aflatoxin B1 and Ochratoxin A in Wistar Rats

**DOI:** 10.3390/molecules30030647

**Published:** 2025-02-01

**Authors:** Pilar Vila-Donat, Dora Sánchez, Lara Manyes, Alessandra Cimbalo

**Affiliations:** Biotech Agrifood Lab, Faculty of Pharmacy and Food Sciences, University of Valencia, Avda. Vicent Andrés Estellés s/n, 46100 Burjassot, Spain; pilar.vila@uv.es (P.V.-D.); dora.sanchez@espam.edu.ec (D.S.); alessandra.cimbalo@uv.es (A.C.)

**Keywords:** mycotoxin excretion, feces, pumpkin, fermented whey, in vivo

## Abstract

This study evaluates the effects of fermented whey (FW) and pumpkin (P) on the excretion of aflatoxin B1 (AFB1) and ochratoxin A (OTA) in rats using immunoaffinity column cleanup and high-performance liquid chromatography–fluorescence detection (IAC-LC-FLD). The method achieved detection limits of 0.1 µg/kg for AFB1 and 0.3 µg/kg for OTA, with recovery rates ranging from 72–92% for AFB1 and 88–98% for OTA. A fecal analysis of 100 rats showed peak AFB1 concentrations of 418 µg/kg and OTA of 1729 µg/kg. In the toxin-exposed groups, OTA levels were higher than AFB1, with males in the OTA-only group showing significantly higher OTA (1729 ± 712 µg/kg) than females (933 ± 512 µg/kg). In the AFB1-only group, the fecal levels were 52 ± 61 µg/kg in males and 91 ± 77 µg/kg in females. The AFB1 + FW group showed notable AFB1 concentrations (211 ± 51 µg/kg in males, 230 ± 36 µg/kg in females). The FW + P combination further influenced excretion, with higher AFB1 and OTA levels. These findings suggest that FW and P modulate mycotoxin excretion and may play a role in mycotoxin detoxification, providing insight into dietary strategies to reduce mycotoxin exposure and its harmful effects.

## 1. Introduction

Aflatoxin B1 (AFB1) and ochratoxin A (OTA) are two highly toxic mycotoxins produced by fungi, commonly contaminating food and posing significant health risks to both humans and animals [1]. AFB1 is primarily produced by *Aspergillus flavus* (*A. flavus*) and *A. parasiticus*, while OTA is produced by species like *A. ochraceus* and *Penicillium verrucosum*. These toxins are known for their carcinogenic, nephrotoxic, and immunosuppressive properties, making their elimination from the body critical for reducing their adverse effects. These mycotoxins contaminate various food products, including cereals, nuts, dried fruits, and animal feeds. AFB1 is highly carcinogenic, and OTA is known for its nephrotoxic effects. The International Agency for Research on Cancer (IARC) classifies AFB1 as a Group 1 human carcinogen, primarily affecting the liver, while OTA is categorized as a Group 2B possible carcinogen, associated with kidney toxicity and other systemic effects [2]. Moreover, AFB1 causes considerable damage to various tissues, including the central nervous system [3].

Exposure to AFB1 and OTA has been linked to various adverse health outcomes, such as liver cancer, kidney damage, immune suppression, and developmental toxicity. These mycotoxins are metabolized in the liver and kidneys, leading to the formation of reactive intermediates that bind to DNA and proteins and cause oxidative stress, mutations, and cell damage [4]. In animals, particularly livestock, chronic exposure results in reduced growth rates, immunosuppression, and compromised productivity, leading to economic losses in the agricultural sector [5]. Additionally, these toxins can enter the food chain through contaminated animal products, further amplifying the risk to humans.

Given the severe health implications associated with AFB1 and OTA, the World Health Organization (WHO) emphasizes the urgent need for effective prevention and control measures throughout the entire food production chain [6]. This includes monitoring and regulating the conditions under which food is produced, stored, and processed. Understanding and implementing methods to mitigate the toxicity of these mycotoxins is crucial to protect public health and ensure food safety [7]. The addition of functional ingredients from various food matrices rich in bioactive compounds has been previously investigated to assess the mycotoxins’ bioaccessibility reduction [7,8,9].

Bioactive ingredients, including dietary fibers, polyphenols, and probiotics, have been increasingly studied for their potential role in detoxifying harmful substances [9,10]. These compounds may exert protective effects by binding to toxins in the gastrointestinal tract, enhancing fecal excretion, or altering toxin metabolism, thereby reducing the risk of absorption and systemic toxicity [11]. Lactic acid bacteria (LAB) are at the top of the list of microorganisms for the degradation of mycotoxins [12]. Fermented whey (FW) contains probiotics like *Lactobacillus* and *Bifidobacterium* strains, which have been demonstrated to enhance gut health by improving microbiota balance and gut barrier function [13]. This activity can reduce the absorption of harmful substances, including mycotoxins, through mechanisms like toxin binding and bioavailability modulation [14]. Studies show that probiotics, including those in FW, can detoxify mycotoxins and promote beneficial microbiota to counteract gut dysbiosis [15].

On the other hand, pumpkin (P) is recognized for its high carotenoid content, particularly β-carotene, which provides strong antioxidant and anti-inflammatory properties [16]. These compounds mitigate the oxidative stress induced by toxins and may indirectly aid in detoxification. Pumpkin’s high fiber content also supports gut health by promoting regular bowel movements and facilitating toxin elimination [7]. Animal studies, such as Wistar rats, serve as a model for investigating the effectiveness of bioactive components in the mitigation of the harmful effects of mycotoxins. In fact, previous authors reported that the inclusion of FW and P in the diet influenced the urinary excretion of AFB1 and OTA, and this effect varied depending on the sex of the rats, the type of mycotoxin, and the exposure dosage [17].

The aim of this study is to evaluate the effects of FW and P, both individually and in combination, on the fecal excretion of two mycotoxins: AFB1 and OTA. Specifically, it seeks to determine whether these bioactive components, known for their probiotic, antioxidant, and detoxifying properties, enhance the elimination of these harmful substances through feces in a Wistar rat model. This investigation addresses the potential synergistic detoxification effects of FW and P when administered together and their implications for reducing toxin bioavailability in the gastrointestinal tract. By investigating how these natural compounds modulate the fecal excretion of AFB1 and OTA, the research could reveal promising dietary interventions or therapeutic strategies that reduce the systemic absorption and toxicity of these mycotoxins.

Such findings may offer valuable knowledge into the development of functional foods or supplements designed to enhance detoxification processes. These interventions could play a significant role in minimizing the health risks posed by AFB1 and OTA exposure, particularly in areas where mycotoxin contamination of food is prevalent. In the end, this research contributes to broader efforts to improve food safety and public health by identifying natural, cost-effective solutions to mitigate the toxic effects of mycotoxins in both animals and humans.

## 2. Results and Discussion

The extraction methods of feces were performed using a clean-up process performed using AflaOchra IAC and based on the previous work of Rodrigues et al. [18], with slight modifications and highlighted in Table 1 below, leading to five protocols (M1, M2, M3, M4, and M5). The modifications in methods (M1–M5) involved different homogenization processes (e.g., equipment and time) and variations in the extraction solution and final supernatant volumes, altering the analyte concentration and extraction efficiency.

### 2.1. Sample Extraction Optimization and Clean-Up

The five protocols (M1–M5) were optimized to ensure adequate recoveries, reduce matrix interferences, and achieve the lowest detection limits (LODs) for this study (Table 2).

The combined IAC cleaning method was tested by analyzing blank feces samples (verified to be free of mycotoxins). Then, these samples were fortified with AFB1 and OTA at the beginning of the extraction process. Recoveries were calculated based on the fortified samples, with all analyses performed in triplicate (*n* = 3) to ensure accuracy and reproducibility (Table 2). Based on the data provided, the optimization of the method across the five protocols yielded a strong linear correlation coefficient (r^2^ > 0.999), with LOD and LOQ (limit of quantification) of 0.1 µg/kg and 0.3 µg/kg, respectively, for both AFB1 and OTA. The recovery rates of the five different methods (M1–M5) were evaluated by introducing a known concentration of 500 µg/kg of the target compound into the sample at the start of the extraction process. This allowed for the assessment of each method’s efficiency in retrieving the spiked compound from the sample matrix. The recovery % varied significantly across methods, with M4 achieving the highest recoveries: 70% ± 2.5% for AFB1 and 110% ± 0.1% for OTA. Conversely, the other methods demonstrated lower recovery rates for AFB1, ranging from 48% to 61%, as follows: M1: 61% ± 1.6%, M2: 48% ± 0.1%, M3: 55% ± 0.1%, and M5: 59% ± 1.5%. As for OTA, recoveries for the other methods ranged from 76% to 106%. For instance, M1 recovery was 88% ±1%, and for M2, it was 86% ±0.1%. For M3, it was 76% ±0.6%, and for the recovery obtained by M5, it was 106% ±0.8% (Table 2). These results highlight M4 as the most efficient for simultaneous AFB1 and OTA recovery. However, the differences between the five methods were not solely due to homogenization but also to the volume of extraction solution used—20 mL in M4 and M5 and 10 mL in the other methods—as well as the volume of supernatant, which was 2 mL for M1, M2, and M4 and 4 mL for M3 and M5. Therefore, the better recoveries observed for M4 were due to the combination of homogenization (45 min magnetic stirring), the volume of extraction solution (20 mL), and the volume of supernatant (2 mL). Method M4 involved mixing 1 g of the sample with 20 mL of 80% MeOH. The mixture was stirred for 45 min on a digital magnetic plate, followed by centrifugation at 4000 rpm for 10 min. Then, 10 mL of PBS were added to 2 mL of the supernatant, and the resulting buffered solution was purified using the AflaOchra IAC. The combined IAC method for the simultaneous extraction of AFB1 and OTA yielded acceptable recoveries, demonstrating that this approach does not significantly compromise the recovery efficiency of either mycotoxin.

The results mentioned above are consistent with those presented by Krausová et al. [19] in their research on mycotoxins in infant feces, where a multi-mycotoxin analytical method was validated using polytetrafluoroethylene filters for extraction and subsequent analysis by LC-MS/MS, achieving recoveries of AFB1 between 79 and 84% (±18% RSD) and recoveries of OTA between 105 and 132% (±8% RSD) in that matrix.

On the other hand, in the study conducted by Lauwers et al. [20] a multi-method approach was developed and validated to measure both mycotoxins and their metabolites in various biological matrices from pigs and broiler chickens. LSE (liquid–solid extraction) was used for mycotoxin analysis in pig feces, and MeOH, ACN, and ethyl acetate were initially evaluated as solvents. However, they did not provide satisfactory recovery results, and the extracts were not clean enough to be injected into the LC-MS/MS instrument. Therefore, they also tested other extraction solvents (acetone and diethyl ether), but the recovery of mycotoxins was still insufficient due to the complexity of the matrix. Finally, they addressed this challenge by testing different solvent combinations and combining the solvents with solid-phase extraction (SPE) columns, achieving acceptable results (≥60%) for most of the mycotoxins (59% for AFB1 and 54% for OTA in pig feces, and 64% for AFB1 and 68% for OTA in chicken excreta). This indicates that, due to the complexity of these matrices, purification through columns is necessary to achieve better recoveries. These results are in line with those of Streit et al. [21], who found that the recoveries of OTA in pig feces ranged from 90–98%, with an RSD of 3.7%, using LC-MS/MS. Similarly, Jurišić et al. [22] achieved a recovery of 98% of AFB1 in chicken excreta by extraction with ACN/H_2_O/CH_3_COOH, Strata C18-T cartridges for clean-up, and analysis using LC-MS/MS.

### 2.2. Validation Results

The M4 was successfully validated, showing acceptable recoveries, precision, and linearity that align with the European Commission legislation on analytical method performance [23]. This legislation specifies recovery limits of 70–120% and relative standard deviations (RSD) of <20%.

The linear range for the matrix calibration curve was established using eight concentration points spanning 5–1500 µg/kg for both AFB1 and OTA. The correlation coefficients (r^2^) were consistently greater than 0.999, indicating excellent linearity. The LOD and LOQ were determined to be 0.1 µg/kg and 0.3 µg/kg, respectively, for both mycotoxins (Table 3).

For method M4, recovery rates were tested at three concentrations (250, 500, and 1000 µg/kg), demonstrating recovery rates of 72–92% (RSD ±7.5%) for AFB1 and 88–98% (RSD ±2%) for OTA (Table 3). These results confirm that M4 meets the necessary criteria for reliable and accurate mycotoxin analysis.

### 2.3. Determination of Mycotoxins in Feces Samples

After validation, the M4 extraction method was applied to analyze 100 fecal samples collected from Wistar rats (both males and females) fed with a diet contaminated with AFB1 and OTA and supplemented with 1% bioactive compounds (FW and P) for 28 days. Due to insufficient defecation, 20 samples could not be collected for analysis. The study aimed to evaluate AFB1 and OTA levels in the fecal samples under different conditions, including various groups with and without FW and P supplementation. The data, which includes concentrations of both mycotoxins, were categorized based on gender (males and females) and the different exposures administered, as outlined in Table 4.

Based on the data in Table 4, both male and female rats in the control groups (non-contaminated) showed no detectable levels of AFB1 or OTA (<LOD). The analysis indicated that the OTA concentrations in feces were consistently higher than those of AFB1 in both genders. In females, the AFB1 levels ranged from 91 to 240 µg/kg, while the OTA levels ranged from 194 to 1499 µg/kg. For males, the AFB1 concentrations ranged from 52 to 418 µg/kg, and the OTA levels ranged from 702 to 1729 µg/kg.

In the AFB1 group, the feces from male rats showed an AFB1 concentration of 52 ± 61 µg/kg, whereas the female feces had higher levels (91 ± 77 µg/kg), with OTA undetectable in these groups. In the OTA group, the OTA levels were significantly higher in both genders, with the male feces having 1729 ± 712 µg/kg and the female feces 933 ± 512 µg/kg and no detectable AFB1. The combined exposure group (AFB1 + OTA) showed moderate concentrations of both toxins, with the male feces having 157 ± 109 µg/kg AFB1 and 840 ± 472 µg/kg OTA and the female feces having 94 ± 37 µg/kg AFB1 and 1384 ± 419 µg/kg OTA (Table 4).

In the FW groups, the addition of FW with either AFB1 or OTA influenced the mycotoxin levels. For example, in the AFB1 + FW group, both males and females exhibited significant AFB1 concentrations (211 ± 51 µg/kg for males and 230 ± 36 µg/kg for females), with no detectable OTA. The OTA + FW group showed notable OTA levels in females (194 ± 167), whereas males had higher levels (1305 ± 40 µg/kg) (Table 4). However, this difference could be attributed to the smaller sample size in the OTA + FW group, as only two out of five rats had fecal samples available for analysis, compared to the typical five samples per group for both males and females. This limited number of samples may have been insufficient to establish a statistically significant difference.

When both bioactive ingredients, FW and P, were combined, the results were interesting. In the AFB1 + FW + P group, male feces showed AFB1 levels of 418 ± 204 µg/kg, and females, 240 ± 8 µg/kg, with no detectable OTA. In the OTA + FW + P group, the OTA levels were substantial, with males showing 1651 ± 419 µg/kg and females 1499 ± 876 µg/kg. Finally, the AFB1 + OTA + FW + P group showed moderate levels of both AFB1 and OTA, with the male and female rats exhibiting approximately similar concentrations of both toxins (Table 4). These findings suggest that the combination of FW and P could influence the absorption or excretion of mycotoxins in rats. The data highlight the potential role of FW and P in the modulation of mycotoxin concentrations, but further analysis is needed to understand the underlying mechanisms and the effects of these interventions.

### 2.4. Effect of Bioactive Ingredients on Fecal Excretion of Mycotoxins

**Figure 1:**
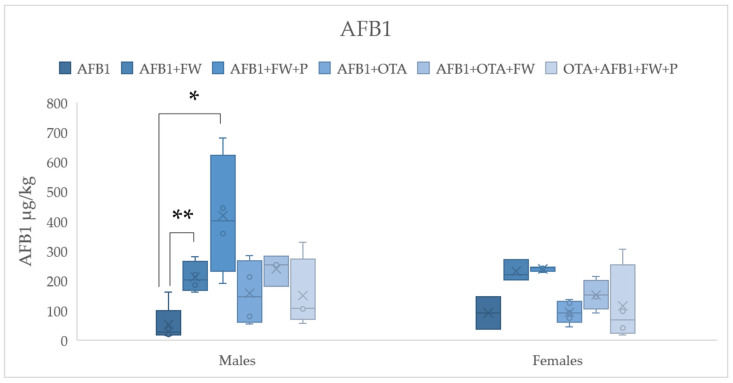
Effects of fermented whey (FW) and pumpkin (P) on fecal aflatoxin B1 (AFB1) levels in male and female Wistar rats. (*) indicates statistically significant differences (*p* ≤ 0.05) in AFB1 fecal levels between experimental groups. (**) indicates statistically significant differences (*p* ≤ 0.01) in AFB1 fecal levels between experimental groups.

**Figure 2:**
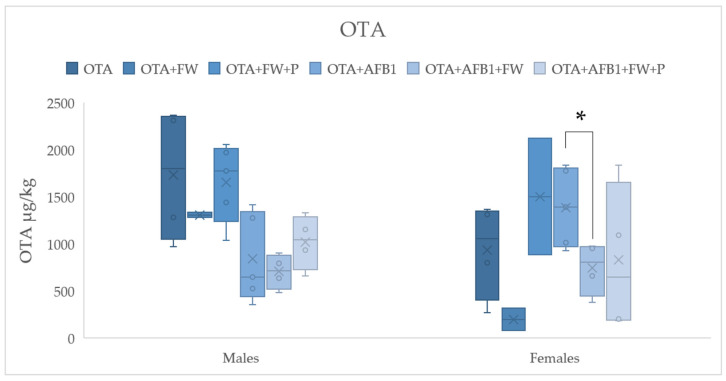
Effects of fermented whey (FW) and pumpkin (P) on ochratoxin A (OTA) fecal levels in male and female Wistar rats. (*) indicates statistically significant differences (*p* ≤ 0.05) in OTA fecal levels between experimental groups.

To assess how bioactive ingredients affect the excretion ratios of AFB1 and OTA in feces, all exposure groups (except the mycotoxin-free controls) were compared. The analysis involved the use of a Student’s *t*-test to examine the differences between pairs of conditions, which included either AFB1 or OTA alone or the combination of both (AFB1 + OTA) in male and female rats. The results, shown in Figure 1 and Figure 2, allowed for the determination of any significant changes in mycotoxin excretion due to the presence of bioactive compounds. This statistical approach helps highlight how these bioactive ingredients may influence the elimination of the mycotoxins, contributing to the understanding of their potential detoxification properties.

#### 2.4.1. Effects of Bioactive Compounds on AFB1 Excretion

The concentration of AFB1 in the feces from the FW+AFB1 group was significantly higher (*p* ≤ 0.01) compared to the control group in males, with a similar trend observed in females. Similarly, fecal AFB1 concentrations in the FW+P+AFB1 group were significantly higher (*p* ≤ 0.05) than those in the control group in males, with a comparable trend in females. The concentration of AFB1 in the feces from the FW + AFB1 group was significantly higher (*p* ≤ 0.01) compared to the control group in males, with a similar trend observed in females. Similarly, fecal AFB1 concentrations in the FW + P + AFB1 group were significantly higher (*p* ≤ 0.05) than those in the control group in males, with a comparable trend in females (Figure 1). These findings suggest that the addition of FW and P to the diet may enhance the retention or absorption of AFB1, potentially due to interactions between these bioactive compounds and the mycotoxin in the digestive system. Similar results have been observed in other studies, where dietary interventions with bioactive compounds influenced mycotoxin metabolism and excretion (Table 5) [17,24].

#### 2.4.2. Effects of Bioactive Compounds on OTA Excretion

In females, the concentration of OTA in feces from the FW + AFB1 + OTA group was significantly lower (*p* ≤ 0.05) compared to the AFB1 + OTA group, with a similar trend observed in males (Figure 2). This suggests that the combination of FW with AFB1 and OTA may have had a protective effect, reducing the fecal excretion of OTA. Such findings align with previous studies that have indicated the potential of bioactive compounds, like those found in FW, to influence the absorption, metabolism, and excretion of mycotoxins in the body [17,25]. These effects may be due to the ability of the LAB present in FW to modulate gut health, promote detoxification, or alter mycotoxin bioavailability. Further research is needed to explore the underlying mechanisms behind this observation.

These results are consistent with those presented by Tian et al. [24], where the AFB1 content in feces collected from mice, following 12 h of forced feeding with AFB1 and either live or inactivated *Lactobacillus plantarum* T3 showed that fecal AFB1 levels were almost twice as high as those in the unexposed group. This suggests that *L. plantarum* T3 may effectively reduce AFB1 accumulation in the intestine through increased fecal excretion, thereby mitigating its toxic effects on the host (Table 5).

The observed lower excretion of OTA in feces when including FW could be linked to an increased OTA excretion via urine in rats, as noted by Pantaya et al. [25] (Table 5). Their research found that a high-starch diet correlated with decreased fecal OTA excretion and increased urinary excretion of OTA 24 h after exposure. Likewise, the rats exposed to diets with FW and P had a higher urinary excretion of OTA than of AFB1, but the urinary increase of OTA excretion could not be correlated with the reduction in OTA excretion in feces. The addition of bioactive compounds to the diets influenced mycotoxin excretion in urine, with effects varying depending on the rat’s sex, the type of mycotoxin (individual or combined), and the exposure dosage [17]. 

To further understand the effects of bioactive compounds, it is important to study both mycotoxins and their metabolites in target organs. Studies by Rushing and Selim [26] and Mupunga et al. [27] suggest that more than half of the ingested AFB1 is metabolized by the liver, with a smaller amount metabolized by the kidneys. Additionally, unmetabolized AFB1 is excreted primarily via feces. Likewise, individual responses to exposure to AFs related to the dose should be considered, as these effects can be influenced by the host, the environment, and genetic factors.

In contrast, OTA is predominantly metabolized by the kidneys in rats [28]. The OTA molecules are more stable and soluble and are primarily excreted via the renal route [29]. According to Vidal et al. [30], the amount of OTA absorbed depends on the species, and the results show a high level of variability. Free OTA is more frequently found in urine and feces than its metabolites, due to the low level of metabolism. This assessment of multiple organs and excretion pathways would offer a more thorough understanding of how bioactive compounds impact mycotoxin dynamics in the body.

## 3. Material and Methods

### 3.1. Standards and Solutions

OTA and AFB1 standards were obtained from Sigma-Aldrich (St. Louis, MO, USA), with a purity of ≥98% (HPLC grade). Stock solutions of the mycotoxins were individually prepared in methanol (MeOH) at a concentration of 100 µg/mL, followed by the preparation of serial dilutions. All working solutions were stored at −20 °C and shielded from light to maintain stability.

### 3.2. Chemical and Reagents

LC-grade ACN and MeOH were procured from Fisher Scientific (Loughborough, UK). Ultrapure water with a resistivity of <18.2 MΩ·cm was produced in the laboratory using a Milli-QSP^®^ Reagent Water System (Millipore, Bedford, MA, USA). Glacial acetic acid (CH_3_COOH, >99% purity) was also sourced from Fisher Scientific (Loughborough, UK), while phosphate-buffered saline (PBS) tablets were obtained from Fisher Scientific (Belgium, UK).

### 3.3. In Vivo Study Design

The current study builds upon the experimental framework established by Vila-Donat et al. [17], investigating the effects of bioactive dietary ingredients on the urinary excretion of AFB1 and OTA in Wistar rats. The animals were housed under standard laboratory conditions (regulated temperature, humidity, and a 12 h light/dark cycle) and were provided ad libitum access to water and the assigned diets. Ethical guidelines for animal welfare were strictly followed throughout the experiment.

The study involved 120 rats (60 males and 60 females), divided into 12 groups, with each assigned a tailored diet. The diets included control feeds and feeds contaminated with AFB1 and OTA, which were prepared using grains inoculated with mycotoxin-producing fungi (*A. flavus* for AFB1 and *A steynii* for OTA). In addition, bioactive dietary ingredients, such as 1% P and 1% FW, were supplemented in specific diets to evaluate their impact on the fecal excretion of mycotoxins.

Fecal samples were periodically collected over the 28-day study period to measure AFB1 and OTA levels, facilitating the assessment of the effects of dietary interventions on mycotoxin elimination. Mycotoxin quantification in the feeds was conducted using LSE and LC-FLD methods. The detailed experimental procedures, including feed preparation recipes and the specific concentrations of mycotoxins in the feeds, have been previously detailed in Vila-Donat et al. [17].

### 3.4. Collection of Fecal Samples

**Figure 3:**
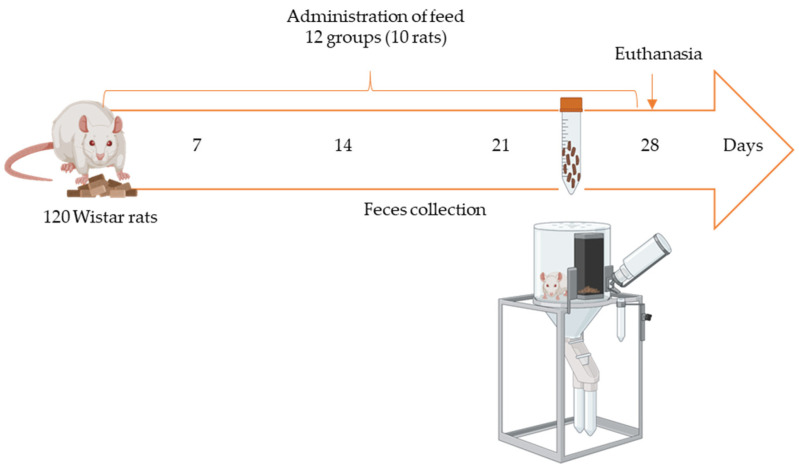
In vivo study scheme.

Fecal samples were gathered weekly from each animal (Figure 3), with each one housed individually in a metabolic cage for 24 h following the start of feed exposure. The collected fecal samples were stored frozen at −20 °C in Falcon tubes. The blank feces used for method validation were obtained from rats fed the control diet after confirming the absence of mycotoxins through analysis.

Once the exposure time had elapsed (28 days), the rats were sacrificed with isoflurane by inhalation, and the organs (liver, kidneys, etc.) were stored at −80 °C.

### 3.5. Extraction of AFB1 and OTA from Feces

The extraction of mycotoxins from feces was carried out using a clean-up process with the AflaOchra IAC, as described by Rodrigues et al. (2019) with slight modifications. The feces samples were first thawed and homogenized by grinding, and then, 1 g was mixed with 20 mL of 80% MeOH. The mixture was stirred for 45 min on a digital magnetic plate, followed by centrifugation at 4000 rpm for 10 min. Afterward, 10 mL of PBS were added to 2 mL of the supernatant, and the resulting buffered solution was purified using the AflaOchra IAC. For extraction, a vacuum-based SPE system was used, designed to concentrate, purify, or isolate analytes from complex matrices. Key components include a glass chamber and waste container, a position cover with luer adapters and a seal, luer connectors, stopcocks, guide needles, posts, shelves, a pressure gauge, and a mounting valve. The system accommodates 12 or 24 samples, ensuring efficient and precise sample preparation.

**Figure 4:**
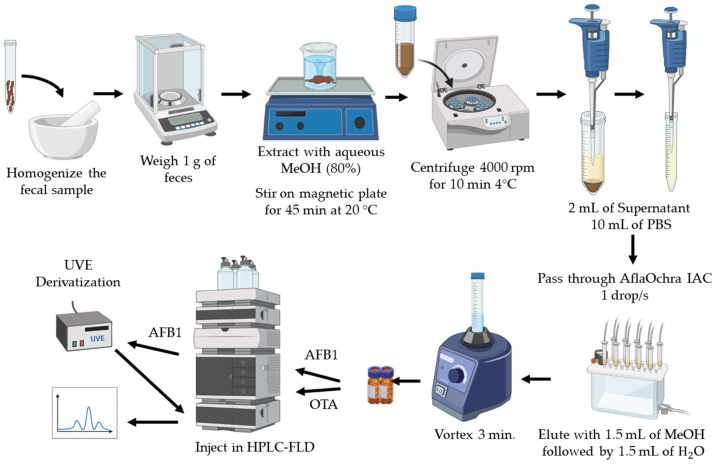
Extraction method of aflatoxin B1 (AFB1) and ochratoxin A (OTA) in feces using AflaOchra immunoaffinity columns (IACs) and analysis via liquid chromatography with fluorescence detection (LC-FLD).

The column setup included a 10 mL syringe on top, an adapter, and a stopcock at the bottom to regulate the flow at 1 drop per second. Buffered samples were loaded into these columns. Then, the columns were washed, and finally, the elution of the compounds of interest was performed. AFB1 and OTA were eluted using a 1.5 mL mixture of MeOH and H_2_O (1:1, *v*/*v*) and collected in a glass vial. After the elution, air is passed through the system using the glass vacuum collector to ensure that all residual eluate is completely removed from the IAC columns. The extracted samples were then transferred to vials and directly injected into the LC-FLD system, as described in the following sections (Figure 4).

The percentage of humidity in the samples was determined after mycotoxin analysis to avoid potential degradation of the mycotoxins due to heating and logistical reasons. Subsequently, the mycotoxin concentration values were adjusted, and the results were expressed based on dry feces (Table 4).

The procedure involved oven drying, where wet samples were weighed in porcelain capsules and dried at 100 °C for 8 h until a constant weight was achieved. The humidity percentage was then calculated based on the weight loss during the drying process using the following formula:

$$Humidity\;(\%)=\frac{\left(W1-W2\right)}{W1}\times 100$$

W1= initial weight (g) of the sample (before drying).

W2= final weight (g) of the sample (after drying).

### 3.6. Validation Methodology

The analytical procedures for AFB1 and OTA were validated by assessing the key performance metrics of selectivity, linearity, precision (intra- and inter-day variability), recovery, LOD, and LOQ. Linearity, sensitivity, and recoveries were evaluated for feces in compliance with European Decision 2002/657/EC [23].

Blank samples were obtained from 10 animals (5 males and 5 females) to serve as uncontaminated reference material. These animals were not exposed to the contaminated diet, ensuring that the samples were suitable for comparing mycotoxin levels and verifying the absence of contamination in the control group. To determine the method’s sensitivity, LOD and LOQ were established based on signal-to-noise ratios (S/Ns) of ≥3 and ≥10, respectively. These metrics were assessed for both the standard solutions and the spiked samples, which were processed identically to the feeding study samples. Method precision was evaluated by calculating the RSD for repeatability. Linearity was examined by preparing and analyzing the calibration curves for each mycotoxin, establishing the correlation between analyte concentration and its corresponding response. Calibration points were generated by diluting AFB1 or OTA from an initial concentration of 10 μg/mL in MeOH with MeOH/H_2_O (1:1, *v*/*v*) to create at least five data points.

Matrix-matched calibration curves were developed by spiking blank feces samples with AFB1 and OTA standards (10 μg/mL in MeOH) at various concentrations. These calibration points spanned a minimum of eight levels, ranging from 5 to 1500 μg/kg (Table 3).

For recovery analysis, rat feces samples were fortified with known concentrations of AFB1 and OTA standards (250, 500, and 1000 μg/kg) prior to the extraction process. The response from the fortified samples was compared to that of the blank samples, with blank areas subtracted from the spiked areas. Recovery percentages were calculated using the formula:

$$Recovery\;\%=\left(\frac{Theoretical\;concentration}{Experimental\;concentration}\right)\times 100$$

The validation results for the quantitative determination method of AFB1 and OTA, including the linear regression equation, linearity range, regression coefficients, LOD/LOQ, and recovery percentages, are summarized in Table 3.

### 3.7. LC-FLD Analysis

AFB1 and OTA quantifications were conducted using liquid chromatography (LC) on an Agilent 1100 series device (Agilent Technologies, Santa Clara, CA, USA) outfitted with an autosampler, degasser, quaternary pump, and Agilent 1200 FLD detector (Agilent Technologies, Santa Clara, CA, USA). Data analysis was performed using the Agilent Open Lab CDS ChemStation Edition software (rev. C.01.10, version 3.2.23). A UVE™ photochemical reactor (LC Tech, Jasco Analítico S.L., Madrid, Spain) was installed between the analytical column and the FLD detector to boost the fluorescence signal of AFB1. Chromatographic separation utilized a reversed-phase column (150 mm × 4.6 mm, 100 A, 5 μm particle size) H17-382064 5720-0076 (Phenomenex, Palo Alto, CA, USA), maintained at 40 °C.

For AFB1 analysis, the mobile phase in isocratic mode comprised H_2_O/ACN/MeOH (60/10/30 *v*/*v*), with a flow rate of 1 mL/min. The excitation and emission wavelengths were adjusted to 365 nm and 440 nm, respectively. In the case of OTA analysis, the mobile phase included ACN/H_2_O/CH_3_COOH (55/43/2 *v*/*v*) at a flow rate of 0.8 mL/min, also under isocratic conditions. The excitation and emission wavelengths for OTA were set at 330 nm and 460 nm, respectively. The injection volume was fixed at 40 μL. To ensure that the instrument operated correctly and parameters remained accurate, daily injections of AFB1 and OTA standards with known concentrations were conducted before analyzing the samples.

### 3.8. Statistical Analysis

A statistical evaluation of the data, including correlation analysis, multiple linear regression analysis, and Student’s t-test, was carried out using Microsoft Excel software (version 2019). Comparisons between the control and exposed groups were performed using Student’s *t*-test. A *p*-value ≤ 0.05 was deemed statistically significant. For normal distribution analysis, the Kolmogorov–Smirnov test was applied, and the two-way ANOVA test was followed by two post hoc tests (Tukey and Bonferroni) using IBM SPSS Statistics software (version 29.0.2.0) (Supplementary Materials).

## 4. Conclusions

This study highlights the potential of bioactive compounds like P and FW in modulating the excretion of mycotoxins, specifically AFB1 and OTA, in Wistar rats. The observed effects on toxin bioavailability may result from altered absorption dynamics or enhanced excretion processes, potentially driven by mechanisms like physical adsorption or biochemical interactions. While the exact pathways remain to be clarified, these findings open new avenues for using FW and P as dietary interventions to mitigate mycotoxin exposure. Further research is needed to deepen our understanding of these mechanisms and to assess the practical applications of these compounds in mycotoxin detoxification strategies. Moving forward, research should explore the impact of these dietary interventions on gut microbiota, as shifts in the microbial community may influence the body’s ability to metabolize and eliminate mycotoxins, thereby reducing their toxic effects. Understanding these interactions may provide innovative strategies for improving animal health and public safety.

In conclusion, the results suggest that dietary modifications, including the use of bioactive ingredients, could offer promising solutions for managing mycotoxin contamination in agricultural systems and may serve as a crucial strategy to safeguard both food production and public health.

## Figures and Tables

**Table 1:** Parameters variations for the extraction methods of AFB1 and OTA from feces (M1–M5).

Methods	Parameters		
	Homogenization	MeOH (mL)	Supernatant (mL)
M1	Sonication 30 min	10	2
M2	Magnetic plate stirring 45 min	10	2
M3	Magnetic plate stirring 45 min	10	4
M4	Magnetic plate stirring 45 min	20	2
M5	Magnetic plate stirring 45 min	20	4

**Table 2:** Optimization of the extraction method of aflatoxin B1 (AFB1) and ochratoxin A (OTA) in spiked feces (methods M1, M2, M3, M4, and M5).

Mycotoxin	Linear Range (µg/kg)	Matrix Calibration Line	*r* ^2^	Recovery (%) 500 µg/kg ± RSD				
				M1	M2	M3	M4	M5
AFB1	5–500	y = 1.3216x − 2.2279	0.999	61 ± 1.6	48 ± 0.1	55 ± 0.1	70 ± 2.5	59 ± 1.5
OTA	5–500	y = 0.4636x − 0.4364	0.999	88 ± 1	86 ± 0.1	76 ± 0.6	110 ± 0.1	106 ± 0.8

**Table 3:** Validation results of the LC-FLD method for the analysis of mycotoxins in Wistar rat feces (M4).

Mycotoxin	Linear Range (µg/kg)	Matrix Calibration Curve	*r* ^2^	LOD (µg/kg)	LOQ (µg/kg)	Recovery (%) ± RSD (%) (*n* = 3)		
						250 µg/kg	500 µg/kg	1000 µg/kg
AFB1	5–1500	y = 1.2774x + 1.5931	0.999	0.1	0.3	72 ± 3.5	76 ± 7.5	92 ± 0.2
OTA	5–1500	y = 0.4123x − 0.0661	0.999	0.1	0.3	98 ± 2	88 ± 1.1	98 ± 0.5

LOD: limit of detection. LOQ: limit of quantification. RSD: relative standard deviation.

**Table 4:** Concentration (µg mycotoxin/kg dry feces) of AFB1 and OTA in rat feces samples collected in the fourth week of the in vivo study (*n* = 100).

	Males				Females	
			(µg mycotoxin/kg dry feces)			
Group Description	N	AFB1	OTA	N	AFB1	OTA
Control	5	<LOD	<LOD	5	<LOD	<LOD
AFB1	5	52 ± 61	<LOD	3	91 ± 77	<LOD
OTA	4	<LOD	1729 ± 712	4	<LOD	933 ± 512
AFB1 + OTA	5	157 ± 109	840 ± 472	5	94 ± 37	1384 ± 419
Control: FW	5	<LOD	<LOD	5	<LOD	<LOD
AFB1 + FW	4	211 ± 51	<LOD	3	230 ± 36	<LOD
OTA + FW	2	<LOD	1305 ± 40	2	<LOD	194 ± 167
AFB1 + OTA + FW	4	238 ± 52	702 ± 184	4	152 ± 50	739 ± 282
Control: FW + P	5	<LOD	<LOD	5	<LOD	<LOD
AFB1 + FW + P	4	418 ± 204	<LOD	5	240 ± 8	<LOD
OTA + FW + P	5	10 ± 18	1651 ± 419	2	7 ± 9	1499 ± 876
AFB1 + OTA + FW + P	4	149 ± 121	1017 ± 289	5	115 ± 131	825 ± 794

AFB1: aflatoxin B1. FW: fermented whey. LOD: limit of detection. OTA: ochratoxin A. P: pumpkin^.^

**Table 5:** Comparison of mycotoxin mitigation results: this study vs. other reports.

Bioactive Compounds	Target Mycotoxins	Model Used	Mechanism of Action	Outcome	Practical Application	References
Pumpkin and fermented whey	AFB1 and OTA	Wistar rats	Modulated mycotoxin excretion in feces with increased AFB1 and OTA excretion	Increased AFB1 and OTA excretion in feces; males showed higher OTA excretion than females. Combination of fermented whey and pumpkin influenced the excretion levels significantly	Feed additives for mycotoxin detoxification	This study
Voghiera garlic	AFB1	Caco-2 and Jurkat T cells	Reduced AFB1 bioaccessibility, increased cell viability, and mitigated AFB1-induced toxicity through decreased ROS and improved cell cycle progression	7–8% reduction in AFB1 bioaccessibility; 9–18% increased cell viability; reduced ROS (16%) and mitochondrial ROS (24%)	Functional ingredient in bread	[7]
Grape pomace	OTA	Caco-2 and Jurkat T cells	Reduced OTA bioaccessibility and oxidative stress, improved cell viability	OTA bioaccessibility reduced by 13% with 2% grape pomace in bread; decreased ROS generation and restored cell death to control level	Functional ingredient in bread	[11]
Bilberries (*Vaccinium myrtillus* L.)	OTA	Caco-2 and Jurkat T cells	Phenolic compounds reduce OTA bioaccessibility, cytotoxicity, and oxidative stress	OTA bioaccessibility reduced by 15% at the intestinal level; 3.7-fold decrease in ROS; improved Caco-2 viability and protection against apoptosis/necrosis (Jurkat)	Functional ingredient in bread	[10]
Carotenoid-loaded nanostructured lipid carriers	OTA	Caco-2 and Jurkat T cells	Nanoencapsulation improves carotenoid delivery; mitigates OTA-induced toxicity by reducing ROS and preserving mitochondrial integrity	No significant effect on OTA bioaccessibility or bioavailability; improved cell viability and decreased ROS production	Novel nanotechnology approach for food safety	[9]
Pumpkin and fermented whey	AFB1 and OTA	Wistar rats	Enhanced urinary excretion of AFB1 and OTA	Highest AFB1 concentrations in rats fed fermented whey-enriched diets; increased urinary OTA excretion; effects influenced by sex, mycotoxin type, and exposure dosage	Analytical method and dietary strategy for mycotoxin detoxification	[17]
*Lactobacillus plantarum* T3	AFB1	Mice	Reduced AFB1 accumulation in the intestine through increased fecal excretion	Lower intestinal AFB1 levels	Probiotic use	[24]
High-starch diet	OTA	Rats	Decreased fecal OTA excretion and increased urinary excretion of OTA	Altered OTA metabolism and excretion patterns	Nutritional strategy	[25]
Clay minerals and inactivated yeast	AFB1	Dairy cows	Reduced AFB1 excretion in milk through binding and sequestration of aflatoxins	Reduced AFB1 concentrations in milk in dairy cow	Feed additives for dairy cows	[18]

## Data Availability

The original contributions presented in the study are included.

## Supplementary Materials

The following supporting information can be downloaded at: https://www.mdpi.com/article/10.3390/molecules30030647/s1. A statistical evaluation of the data, including correlation analysis, multiple linear regression analysis, and Student’s *t*-test.
